# Glucose Biosensor Based on Disposable Activated Carbon Electrodes Modified with Platinum Nanoparticles Electrodeposited on Poly(Azure A)

**DOI:** 10.3390/s20164489

**Published:** 2020-08-11

**Authors:** Francisco Jiménez-Fiérrez, María Isabel González-Sánchez, Rebeca Jiménez-Pérez, Jesús Iniesta, Edelmira Valero

**Affiliations:** 1 Department of Physical Chemistry, Higher Technical School of Industrial Engineering, University of Castilla-La Mancha, Campus Universitario s/n, 02071 Albacete, Spain; Francisco.JFierrez@uclm.es (F.J.-F.); MIsabel.Gonzalez@uclm.es (M.I.G.-S.); Rebeca.Jimenez@uclm.es (R.J.-P.); 2 Department of Physical Chemistry and Institute of Electrochemistry, University of Alicante, 03690 San Vicente del Raspeig, Spain; Jesus.Iniesta@ua.es

**Keywords:** glucose, glucose oxidase, enzymatic biosensor, poly(Azure A), platinum nanoparticles, activated screen-printed carbon electrodes

## Abstract

Herein, a novel electrochemical glucose biosensor based on glucose oxidase (GOx) immobilized on a surface containing platinum nanoparticles (PtNPs) electrodeposited on poly(Azure A) (PAA) previously electropolymerized on activated screen-printed carbon electrodes (GOx-PtNPs-PAA-aSPCEs) is reported. The resulting electrochemical biosensor was validated towards glucose oxidation in real samples and further electrochemical measurement associated with the generated H_2_O_2_. The electrochemical biosensor showed an excellent sensitivity (42.7 μA mM^−1^ cm^−2^), limit of detection (7.6 μM), linear range (20 μM–2.3 mM), and good selectivity towards glucose determination. Furthermore, and most importantly, the detection of glucose was performed at a low potential (0.2 V vs. Ag). The high performance of the electrochemical biosensor was explained through surface exploration using field emission SEM, XPS, and impedance measurements. The electrochemical biosensor was successfully applied to glucose quantification in several real samples (commercial juices and a plant cell culture medium), exhibiting a high accuracy when compared with a classical spectrophotometric method. This electrochemical biosensor can be easily prepared and opens up a good alternative in the development of new sensitive glucose sensors.

## 1. Introduction

Glucose analysis is very important and common, mainly because of the disease diabetes mellitus, produced by an abnormal glucose concentration in the blood and tissues, which affects approximately 150 million people around the world [1]. Furthermore, the quantification of glucose levels is not only important in clinical analysis, but also in other areas such as food production or quality control [2]. Therefore, the development of new analytical methods for the determination of this biomolecule is essential. Conventional methods for the analytical determination of glucose include spectrophotometry [3], HPLC [4], capillary zone electrophoresis [5], infrared spectroscopy [6], chemiluminiscence [7], and electrochemical sensing [8]. Electrochemical enzymatic biosensors are an attractive approach for glucose determination, as they have multiple advantages such as specificity, speed of use, simplicity of construction, accuracy, portability, possibility of miniaturization, and easy operation [9]. In particular, electrochemical glucose biosensors offer advantages such as the specific recognition of glucose, low cost, and rapid quantification [10]. 

Amperometric glucose biosensors are usually categorized into three different generations based on the involved mechanism of the electrochemical reaction [8]. The first generation includes biosensors based on the oxidation of glucose, where the production of hydrogen peroxide (H_2_O_2_) or the decrease in oxygen concentration is electrochemically monitored [11]. In the second generation biosensors, low-molecular weight compounds used as mediators in the process of glucose oxidation are the responsible for signal generation at the electrode surface [12]. Finally, the third generation is based on the direct electron transfer between the enzyme active center and the electrode [13]. The glucose biosensor developed in this work belongs to the first generation, particularly to the electrochemical measurement of H_2_O_2_. The principal drawback of the first generation biosensors refers to H_2_O_2_ sensing at a high potential on most electrode materials, whereby possible interfering components within any biological fluid can additionally contribute to the electrochemical signal [14]. One of the most common ways to overcome the aforementioned problem is the coupled use of horseradish peroxidase, which catalyzes the reduction of H_2_O_2_ [12,15]. This approach also suffers from some disadvantages, such as a higher cost, low stability, and the limited binding of horseradish peroxidase to solid surfaces [16]. For that reason, electrode modification using different cost-effective materials has attracted great research interest, with the aim to decrease the working applied potential, thereby avoiding the necessity of using bi-enzymatic systems. In this regard, it has been found that some nanostructures based on transition metals [14,17,18] and conjugated polymers [19,20,21] have the capability to enhance the electrocatalytic activity towards H_2_O_2_ oxidation. Furthermore, the combination of conjugated polymers and metal nanoparticles has been demonstrated to have a positive effect for developing glucose biosensors. These improved analytical features have been shown, for example, in the immobilization of glucose oxidase (GOx) in an electropolymerized poly(*o*-phenylenediamine) film on a platinum nanoparticles-polyvinylferrocenium modified electrode [22], in the glucose sensor based on Pt nanoparticles/polyaniline hydrogen heterostructures [23], and in the biosensor based on GOx entrapped within a polyaniline layer with gold nanoparticles [24].

Screen-printed electrodes (SPEs) offer important practical advantages over conventional electrodes in terms of mass production, miniaturization, high versatility, and the possibility to design portable electrochemical sensors. One of the most relevant benefits is the easy modification of their working surface to develop different sensors and biosensors. Therefore, pre-treatments and modification protocols of the working electrode are made with the aim to enhance electro-transfer properties and sensitivity towards the compound of interest [18,25]. In particular, an H_2_O_2_-basedpre-treatment (carbon activation) [25,26], the polymerization of poly(Azure A) (PAA) using dodecyl sulphate (DS) as a doping ion [19,27], and the electrodeposition of platinum nanoparticles (PtNPs) [18,28] on screen-printed carbon electrodes (SPCEs) have demonstrated excellent electrochemical properties in the measurement of H_2_O_2_. Recently, our research group published a novel non-enzymatic H_2_O_2_ sensor based on PtNPs electrochemically deposited on a PAA film previously electrogenerated on screen-printed carbon electrodes whose surface was previously activated by H_2_O_2_ (aSPCEs) [29]. The synergy between PAA and PtNPs and the high electrochemical surface obtained allowed for the development of a highly sensitive H_2_O_2_ sensor at a low overpotential, with a very appropriate limit of detection, stability, and reproducibility. A variant of this electrode has also been used in the real-time monitoring of the H_2_O_2_ secreted from living plant cells under different stress conditions, demonstrating the excellent applicability of the modified sensor [30].

In this work, we tackle, for the first time, the development of an amperometric biosensor based on GOx immobilized on PtNPs electrogenerated on PAA that has been previously electropolymerized on an aSPCE (GOx-PtNPs-PAA-aSPCE) for the analysis of glucose. The basic principle of its operation is the enzymatic oxidation of glucose, followed by the electrochemical oxidation of H_2_O_2_ at 0.2 V (vs. Ag). The developed glucose biosensor was validated in terms of sensitivity, limit of detection, linear range, selectivity and interferences, repeatability, reproducibility, and its application to glucose quantification in real samples.

## 2. Materials and Methods

### 2.1. Chemicals and Solutions

L-ascorbic acid (sodium salt), L-ascorbate oxidase spatula from *Cucurbita* species, azure A (AA) chloride (dye content ~80%), citric acid (trisodium salt), chloroplatinic acid hexahydrate (99.9%), L-dehydroascorbic acid, D-(−)-fructose, D-(+)-glucose, β-D-glucose oxidase from *Aspergillus niger*, hydrogen peroxide (35%), D-mannitol, sodium dodecyl sulfate (95%), sodium ferrocyanide decahydrate, sodium phosphate (monobasic and dibasic), sucrose, and urea were purchased from Sigma-Aldrich (Madrid, Spain). Sodium hydroxide was from Merck (Madrid, Spain). Potassium ferrocyanide trihydrate (99.95%) was purchased from Probus. A commercial glucose assay kit based on the enzymatic oxidation of glucose by GOx was acquired from Sigma-Aldrich. This is a bi-enzymatic assay in which glucose is oxidized to gluconic acid by GOx, yielding hydrogen peroxide. Hydrogen peroxide then reacts with *o*-dianisidine in the presence of peroxidase to yield a colored product at an acidic pH, which is measured at 540 nm (ref. no. GAGO20-1KT, https://www.sigmaaldrich.com/catalog/product/sigma/gago20?lang=es&region=ES). 

The real samples tested herein consisted of three different commercial fruit juices and a plant cell culture medium. Commercial fruit juices were purchased from a local supermarket. The plant culture medium (Gamborg’s B-5) was purchased from Sigma-Aldrich. The composition of this plant culture medium is described in the literature [31], where it can be seen that it has no glucose in its composition, although it contains other sugars like sucrose. 

All of the reagents were acquired at their highest available purity and were used without further purification. All of the solutions were prepared using ultrapure deionized water with a resistivity of 18.2 MΩ∙cm (Milli-Q system, Millipore, Watford, UK), and were newly prepared every day.

### 2.2. Preparation of Modified Electrodes

Carbon surface modification was performed on disposable screen-printed carbon electrodes (SPCEs; DRP-110, from Metrohm DropSens), with a geometrical area of 12.6 mm^2^, consisting of a carbon ink working electrode, a carbon ink counter electrode, and a silver ink pseudo-reference electrode. The surface of the working electrodes was modified by the following four steps:

(1) Activation of SPCEs: Commercial SPCEs were activated by performing 12 repetitive cyclic voltammograms between 1 and −1 V in 10 mM H_2_O_2_ in 0.1 M of phosphate buffer (PB) with pH 7 at 10 mV∙s^−1^. Once activated, the electrodes (aSPCEs) were rinsed with deionized water and dried in air. It is noteworthy that this protocol has been improved with respect to our previously published work [25], but here, we obtained a similar electrochemical outcome and sensitivity towards H_2_O_2_ oxidation, with a significant reduction of 1 h in the activation time.
(2) Electropolymerization of azure A: PAA was electrodeposited on the working electrode surface of aSPCEs following the protocol described in the literature [19].
(3) PtNPs electrosynthesis: Platinum nanoparticles were electrogenerated by chronoamperometry at −0.4 V for 900 s in a solution 0.2% H_2_PtCl_6_, as indicated in the literature [18]. The resulting electrodes (PtNPs-PAA-aSPCEs) were rinsed with deionized water, dried, and stored until use.
(4) Glucose oxidase immobilization: 10 µL of an enzyme solution at the indicated concentration in 0.05 M of PB, with pH 7, was drop-cast onto the modified surface of the working electrode for a certain time. When the immobilization time was finished, the remaining solution was removed, and the electrodes were first rinsed with deionized water several times and finally with 0.1 M of PB with pH 7. The electrochemical glucose biosensor was named GOx-PtNPs-PAA-aSPCE.

### 2.3. Electrochemical Measurements

The electrochemical experiments were performed using a bipotentiostat μStat 300 from Dropsens (Asturias, Spain; http://www.dropsens.com/). The electro-analysis of glucose was conducted with chronoamperometry by successive additions of micromolar concentrations of this compound under stirring in 0.1 M PB (pH 7). Unless otherwise indicated, the working electrode potential employed was +0.2 V (vs. Ag). 

The electroactive areas of the electrodes were calculated using the Randles–Sevick equation by performing voltammetric cycles at different scan rates in the presence of 1 mM Na_4_Fe(CN)_6_ in 0.1 of a KCl aqueous solution, previously deoxygenated with nitrogen gas. The diffusion coefficient of the Na_4_Fe(CN)_6_ used was 6.5 × 10^−6^ cm^2^∙s^−1^ [32]. The average surface area of the GOx-PtNPs-PAA-aSPCEs biosensor was 4.8 ± 0.1 mm^2^ (*n* = 3). 

The electrochemical impedance spectroscopy (EIS) measurements were performed in an AUTOLAB PGSTAT128N potentiostat with an EIS analyzer (Eco Chemie B.V., The Netherlands) using NOVA 2.0 software. The EIS was carried out at 0.15 V in 1 mM Na_4_Fe(CN)_6_ and 0.1 M KCl. The SPCEs were polarized for 30 s. Then, a sinusoidal amplitude potential perturbation (5 mV rms) was imposed between 65 kHz and 10 mHz, with five points per decade. 

### 2.4. Physicochemical Measurements

The morphology of the electrodes was analyzed using field emission scanning electron microscopy (FE-SEM, HITACHI S-3000N microscope), working at 30 kV with a Bruker Xflash 3001 X-ray detector for the microanalysis. The XPS experiments were recorded on a K-Alpha Thermo Scientific spectrometer using Al-Kα (1486.6 eV) radiation, monochromatized by a twin crystal monochromator, yielding a focused X-ray spot with a diameter of 400 μm mean radius. The alpha hemispherical analyzer was used as an electron energy analyzer, operating in fixed analyzer transmission mode, with a survey scan pass energy of 200 eV and 40 eV narrow scans. The angle between the X-ray source and the analyzer (magic angle) was 54.7°. Avantage software was used for processing the XPS spectra, with energy values referenced to the C1s peak of the adventitious carbon located at 284.6 eV, and a Shirley-type background.

## 3. Results

### 3.1. Characterization and Optimization of the Electrochemical Performance of the Glucose Biosensor

**Figure 1:**
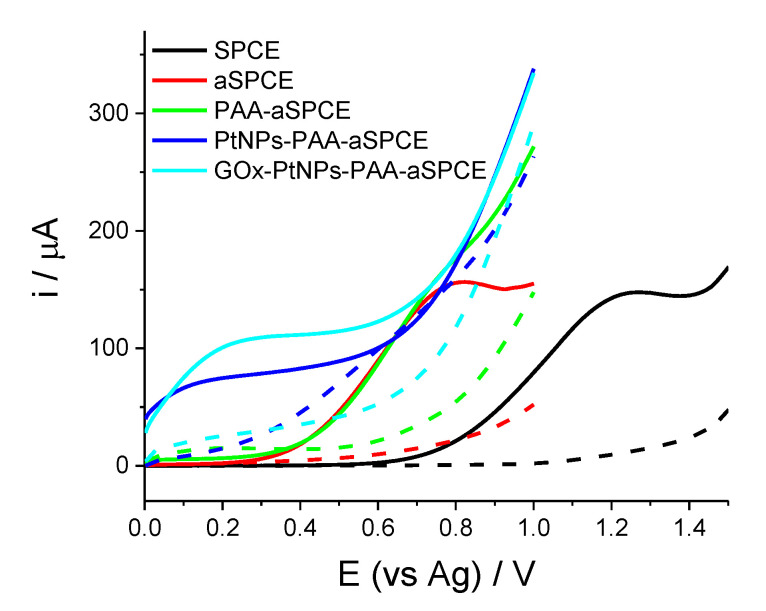
Anodic linear scan voltammetry (LSV) responses of the different electrode modification steps in the absence (dashed lines) and the presence of 5 mM of H_2_O_2_ (solid lines). The measurements were performed in 0.1 M PB.

The electrochemical glucose biosensor developed herein is based on the electrochemical detection of the H_2_O_2_ produced in the oxidation of glucose by the enzyme GOx. Figure 1 shows the linear scan voltammetry (LSV) curves for the H_2_O_2_ electro-oxidation at the different modification steps of the biosensor, both in the absence and presence of H_2_O_2_, namely: SPCE, aSPCE, PAA-aSPCE, PtNPs-PAA-aSPCE, and the final biosensor GOx-PtNPs-PAA-aSPCE.

In the absence of H_2_O_2_, no electroactive peaks were detected by the different working electrodes. In the presence of H_2_O_2_, the electrooxidation peak of this compound appeared at 1.2 V using the pristine (non-activated) electrode; however, the H_2_O_2_ activation of the carbonaceous working electrode (aSPCE) resulted in the displacement of this peak to a potential close to 0.7 V (Figure 1). Accordingly, the electrodeposition of PAA was carried out on the aSPCE [19]. A different behavior for the LSVs was observed, although in the presence of H_2_O_2_, an anodic shoulder was seen at approximately 0.7 V, attributed to the oxidation of H_2_O_2_ (Figure 1, green line). As expected, the electrocatalytic oxidation of H_2_O_2_ was favored with PtNPs-PAA-aSPCE, as depicted in Figure 1 (blue line), with a broad anodic potential centered at 0.2 V. The electrochemical response of GOx-PtNPs-PAA-aSPCE regarding the electrooxidation of H_2_O_2_ is also depicted in Figure 1 (cyan line), and exhibits a broader anodic potential centered at ca. 0.2 V. In summary, this remarkable shift towards lower potentials (from 1.2 V (vs. Ag) in the non-modified electrode to 0.2 V (vs. Ag) in the GOx-PtNPs-PAA-aSPCE) decreases the probability of detecting interfering compounds, and contributes to reducing the signal noise (vide infra).

Before going through the SEM images of the electrodes, the deep exploration of the effect of GOx immobilization on the electrochemical features of the glucose biosensor was addressed, so a brief study of the influence of the enzyme concentration on the enzymatic loading and immobilization time was performed to achieve a biosensor with maximum analytical outcomes for the measurement of glucose. For that, different electrochemical glucose biosensors were fabricated from PtNPs-PAA-aSPCEs by changing the GOx concentration used to be drop-cast on the surface of the working electrode and the time required for enzyme immobilization. Later, all of the electrochemical glucose biosensors were tested for the analytical measurement of glucose by performing a calibration plot (current intensity vs. glucose concentration between 10 and 120 μM) at a potential of 0.2 V. The sensitivity obtained for each particular trial was worked out (Figure S1). The results showed the highest sensitivity for glucose determination when using a concentration of 30 mg∙mL^−1^ GOx (Figure S1A) and an immobilization time of 1 h (Figure S1B). Therefore, these experimental conditions were selected for further experiments and analysis. 

**Figure 2:**
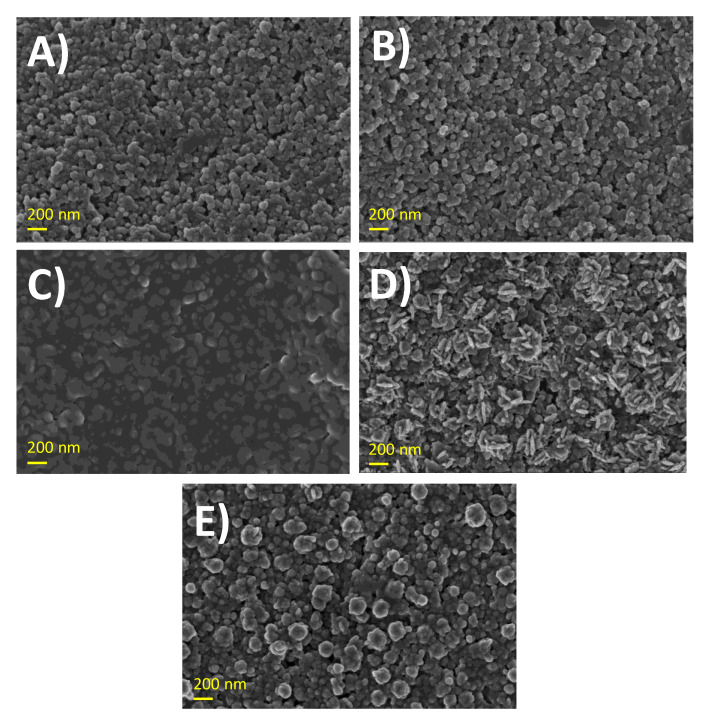
FE-SEM images of: (**A**) screen-printed carbon electrodes (SPCE), (**B**) activated SPCE (aSPCE), (**C**) poly(Azure A) (PAA)-aSPCE, (**D**) platinum nanoparticles (PtNPs)-PAA-aSPCE, and (**E**) glucose oxidase (GOx)-PtNPs-PAA-aSPCEs, with a magnification of 30,000 K.

Figure 2 depicts the FE-SEM images of the surface of the modified electrodes at the different steps. No appreciable differences in the morphology were observed when comparing SPCE and aSPCE (Figure 2A,B), which is a similar response to that reported in the literature [26]. After the electrodeposition of PAA, the surface displayed a smooth morphology, which confirmed that the aSPCE was coated by the polymeric film (Figure 2C). The electrodeposition of PtNPs on PAA (Figure 2D) showed PtNPs with a non-uniform elongated shape (fusiform) with average sizes of 106 ± 17 nm (length) and 33 ± 6 nm (width), which are highly dispersed throughout the PAA film compared with the PtNPs electrodeposited on the naked aSPCE, which provided an ill-defined shape with less distributed and more aggregated PtNPs [30]. It is noteworthy that the characteristic morphology of PtNPs was lost when casting GOx on PtNPs-PAA-aSPCE (Figure 2E), in which distributed small enzyme clusters were observed. These enzyme clusters seemed to cover the platinum nanostructures previously produced.

**Figure 3:**
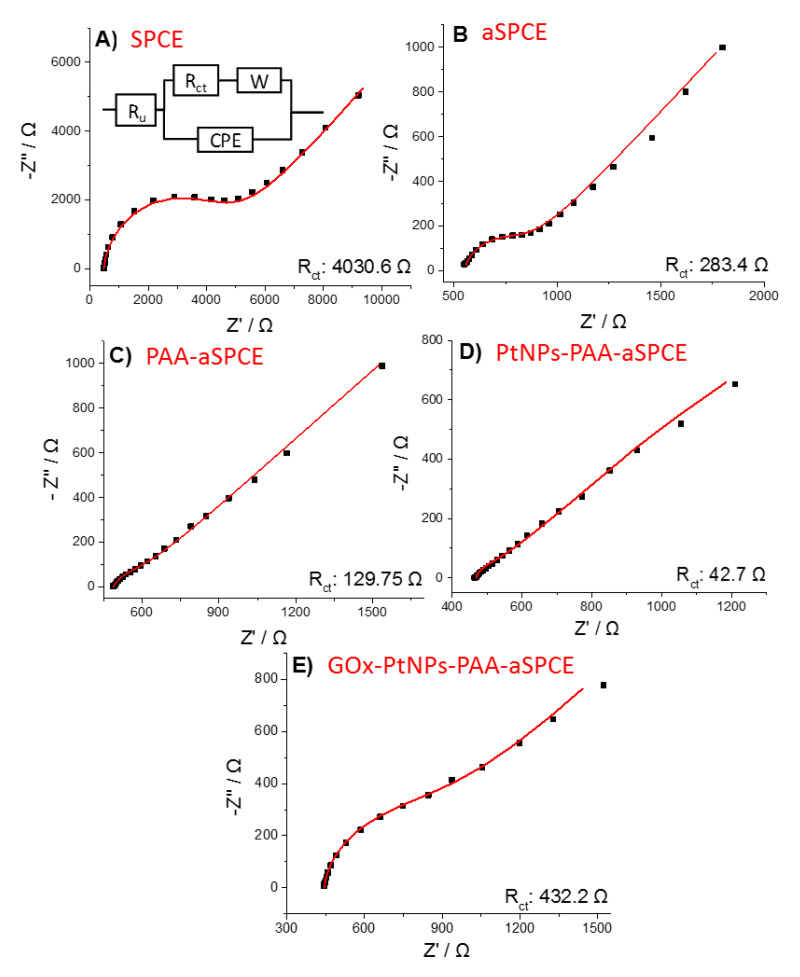
Nyquist plots for the different steps of the working electrode modification: (**A**) SPCE,(**B**) aSPCE, (**C**) PAA-aSPCE, (**D**) PtNPs-PAA-aSPCE and (**E**) GOx-PtNPs-PAA-aSPCE. Electrochemical impedance spectroscopy (EIS) was recorded at 0.15 V (vs. Ag) in 1 mM Na_4_Fe(CN)_6_ (in 0.1 M KCl). Experimental conditions: stabilization time 60 s, amplitude 5 mV, and frequencies 65 kHz–10 mHz, with five points per decade. The inset in (**A**) shows the equivalent circuit used.

The results of Figure 1 have been correlated to the EIS and XPS experiments. The electrochemical response was analyzed using EIS in the presence of an outer sphere redox couple. Figure 3 exhibits the Nyquist diagrams of the EIS experiments, performed in the presence of 1 mM of sodium ferrocyanide by applying a constant potential of 0.15 V (vs. Ag) for (A) SPCE, (B) aSPCE, (C) PAA-aSPCE, (D) PtNPs-PAA-aSPCE, and (E) GOx-PtNPs-PAA-aSPCEs. The inset of Figure 3A shows the Randles’ equivalent model circuit used to fit the experimental data (results shown in Table S1 in Supplementary Material). The semicircles or wide arcs at high frequencies are associated with the charge transfer resistance (*R_ct_*) at the electrode solution interface. This parameter shows the kinetics of the electron transfer of the redox probe at the electrode interface. The *R_ct_* was higher in the SPCEs (4030.6 Ω) because of their low electrical conductivity associated with the carbon ink, which is also reflected on the LSV behavior in Figure 1. The processes of activation, polymerization, and platinization on the working surface considerably reduced *R_ct_*, with values of 283.4, 129.8, and 42.7 Ω, which means an important improvement in the electrical conductivity after these treatments, and therefore, these surface changes are reflected on the electrochemical performance of the glucose biosensor, as depicted in Figure 1, in the absence of H_2_O_2_. Moreover, as expected, the subsequent drop-casting of GOx manifested an increase of *R_ct_* with respect to the PtNPs-PAA-aSPCE electrode (432.2 Ω), although this value is ten times lower than that obtained for the bare SPCE. The increment in *R_ct_* after the addition of the enzyme suggests a hindrance for electron transfer through the dense protein layer of the enzyme [33,34]. This is in agreement with the LSV features of Figure 1, where PtNPs-PAA-aSPCE depicts a less resistive voltammetric behavior compared with that shown for GOx-PtNPs-PAA-aSPCE (in the absence of H_2_O_2_), although the immobilized protein on PtNPs resulted in a slight increase in the current intensity at 0.2 V (after background subtraction).

The electroactive areas obtained for all of the electrodes: SPCE, aSPCE, PAA-aSPCE, PtNPs-PAA-aSPCE, and GOx-PtNPs-PAA-aSPCE are also indicated in Table S1. The results show that carbon activation did not produce any increase in the electroactive area, whereas the coverage with the polymer decreased it and the deposition of the platinum nanoparticles increased it. In addition, a decrease in the electroactive area was also appreciated after the immobilization of GOx, probably due to the coating of the platinum nanostructures previously produced (Figure 2E).

Despite the EIS results in Figure 3E, which denote a more sluggish electron transfer of the whole electrochemical glucose biosensor GOx-PtNPs-PAA-aSPCE, we also explored the LSV response of the biosensor in the presence and absence of glucose (Figure S2, Supplementary Material). Under such conditions, an increase in electrochemical current at low potentials close to 0.2 V was also observed using the GOx-PtNPs-PAA-aSPCE biosensor, in contrast to the poor signal obtained for the previous modified steps of the biosensor, where no significant current was observed in the potential range between 0 and 0.4 V vs. Ag. This fact indicates that glucose can be detected using a potential of 0.2 V (vs. Ag), and that the presence of glucose at high concentrations has no dramatic consequences on the fouling or inactivation of the electrochemical glucose biosensor. Moreover, this is a valuable achievement, as most of published first-generation electrochemical glucose biosensors based on H_2_O_2_ oxidation operate at higher potentials [2,35,36,37,38]. In fact, one of the most critical drawbacks in first generation sensors is the high electrode potential used, where multiple interfering components that might be present in the samples can be oxidized [14].

**Figure 4:**
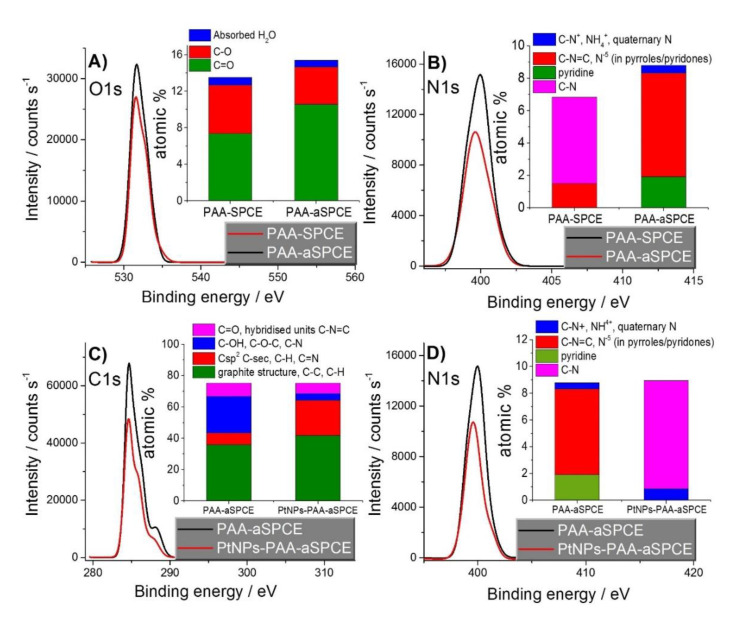
XPS (**A**) O1s and (**B**) N1s spectra for PAA-SPCE and PAA-aSPCE. XPS (**C**) C1s and (**D**) N1s spectra for PAA-aSPCE and PtNPs-PAA-aSPCE. The insets of the respective figures show the atomic surface concentrations of the functional groups after the deconvolution of the peaks.

To further study the electrode surface chemistry of the different electrode modifications, they were characterized using XPS to correlate the electrochemical performance of the different electrodes. First, a comparison between PAA-SPCE and PAA-aSPCE showed the effect of the activation on the electrodeposition feature of the polymer. Figure 4A,B shows the O1s and N1s spectra for PAA-SPCE and PAA-aSPCE, respectively. The deconvolution of the spectra and the assignations of peaks to functional groups [39] are shown in the Supplementary Material (Figure S3). The insets of Figure 4A,B show the functional groups assigned and their atomic percentage (at.%) values. A major content of oxygen-containing functional groups with a higher ratio of C = O/C-O was found in PAA-aSPCE (Figure 4A). This major content in the oxygen-containing functional groups might positively influence the anchorage of the polymer to the working electrode of the aSPCE. Furthermore, the N1s analysis revealed a higher amount (at.%) of nitrogen in PAA-aSPCE (Figure 4B), which could be indicative of a thicker electrodeposited PAA film on the activated carbonaceous surface, with the corresponding increase in the available surface for PtNPs deposition [29]. In addition, the nitrogen species found in PAA-aSPCE are different to those in PAA-SPCE. For example, pyridine nitrogenous species and quaternary nitrogenous charged species were detected in PAA-aSPCE, whereas the C-N was not present, in contrast with PAA-SPCE. The positively charged species could act as active centers for the electrodeposition of PtNPs, as the nanoparticles were synthesized from the negatively charged species. Even though slight differences in the surface chemistry are found between PAA-SPCE and PAA-aSPCE, the coulombic charge during the electropolymerization process was similar (data not shown) and the EIS experiments proved to exhibit a similar Nyquist plot pattern. Nonetheless, the activation of the working electrode surface using H_2_O_2_ was positive in terms of stability and reproducibility [29].

Then, PtNPs-PAA-aSPCE was also analyzed by XPS, and the results were compared to those obtained for PAA-aSPCE (Figure 4C,D). In general, the C1s and N1s spectra showed a decrease in the at.% content of carbon and nitrogen after the deposition of PtNPs. The deconvolution of the C1s and N1s spectra and their assignations to functional groups [39] are shown in the Supplementary Material (Figure S4). The insets of Figure 4C,D show the functional groups assigned and their at.% in each case. In PAA-aSPCE, the energy peak at 285.41 eV was linked to the graphite structure, C-C, C-H, and Csp^2^, whereas the energy peak at 286.14 eV was linked to C-sec, C-H, and C = N [39]. These peaks were slightly shifted to 285.85 and 286.84 eV in PtNPs-PAA-aSPCE, which could be related to the different local bonding environments of the functional groups before and after the electrodeposition of PtNPs [39]. In addition, in PtNPs-PAA-aSPCE, the C-OH, C-O-C, and C-N content decreased, whereas the Csp^2^, C-sec, C-H, and C = N at.% content increased. This might be indicative of the preferential deposition of PtNPs on active sites with C-OH, C-O-C, and C-N. Moreover, the N1s spectra showed a change in the nitrogen species between PAA-aSPCE and PtNPs-PAA-aSPCE, with the disappearance of pyridine nitrogen and the appearance of C-N after the electrodeposition of PtNPs. 

Pt 4f was detected by XPS after the electrodeposition of PtNPs, confirming the presence of platinum on the electrode (data not shown). The Pt 4f_7/2_ peak was deconvoluted in two subpeaks with binding energies of 71.1 and 72 eV, linked to Pt (0) and Pt (OH)_2_ [39]. It was observed that metallic platinum was the major component (>60%, data not shown), which confirms the effective reduction of platinum in the solution and its deposition on PAA-aSPCE.

### 3.2. Analytical Performance of the Glucose Biosensor

**Figure 5:**
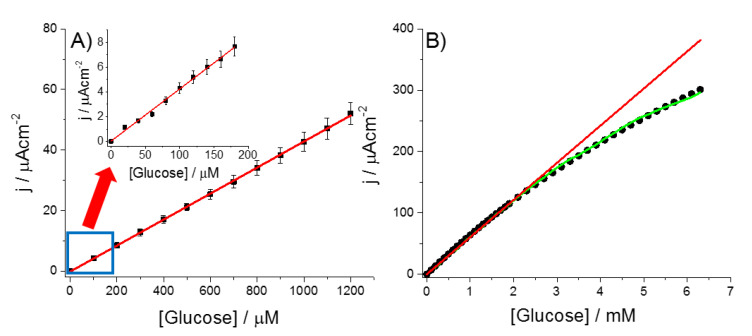
Current outcome obtained by successive additions of glucose in a stirred solution (10 mL) of 100 mM PB, pH 7, at 0.2 V (vs. Ag), using a GOx-PtNPs-PAA-aSPCE. Range of glucose concentration: (**A**) 100−1200 µM; inset of (**A**), 20−200 µM; and (**B**) 0.1−6.5 mM. The experimental data of I vs. time from Figure 5B are shown in Figure S5 (Supplementary Material).

Figure 5 shows the amperometric response of GOx-PtNPs-PAA-aSPCE towards successive additions of glucose in a micromolar (Figure 5A, inset of Figure 5A) and millimolar range (Figure 5B). The electrochemical biosensor showed a sensitivity of 42.7 ± 0.2 μA∙mM^−1^∙cm^−2^, a limit of detection of 7.6 μM (S/N ratio of 3), and a limit of quantification of 25.6 μM (S/N ratio of 10). The estimated linear range was situated between 20 μM and 2.3 mM. The data obtained in Figure 5B were fitted by non-linear regression analysis to a Michaelis–Menten expression (*I = I_max_ [Glucose]/(K_M,app_ + [Glucose])*), obtaining a maximum intensity current response (*I_max_*) and an apparent Michaelis-Menten constant *K_M,app_* of 1002.5 μA · cm^−2^ and 14.5 mM, respectively. The *K_M,app_* obtained was lower than that obtained for glucose oxidase in a bulk solution (33 mM) [40], suggesting that the hybrid nanostructure PtNPs-PAA is a favorable environment for the enzyme. A similar result was reported for GOx immobilized on other metal nanoparticles/conducting polymer composites, such as Pt nanoparticles/polyaniline (PtNPs/PANI) [23], Pt/multi-wall carbon nanotube-polyaniline composite (Pt/MWCNT-PANI) [41], and an electropolymerized poly(*o*-phenylenediamine) film on a PtNPs-polyvinylferrocenium modified electrode [22].

The repeatability of the electrochemical biosensor was calculated by the measurement of 100 µM of glucose using the same electrode (*n* = 10), and was 3.96%. The reproducibility was calculated by performing glucose calibration plots (from 100 to 1200 µM) using three different electrodes, and was estimated to be 4.76%. To check the stability, the electrochemical biosensor was prepared and stored at 4 °C in 100 mM of PB. The biosensor performance was tested at different storage times. The data obtained indicated that the modified electrode exhibited a signal of 83% of the initial sensitivity one day after its preparation, approximately 50% of the initial value after 7 days of storage, and around 30% after 15 days of storage (Figure S6, Supplementary Material). It is far more frequent that the biological component of enzymatic biosensors suffer a loss of activity, principally because of the denaturation and deactivation of the protein, which ultimately reduces the lifetime of the biosensor [42,43]. For that reason, it is always advisable that the immobilization of the biological component (last step in the construction of the biosensor) is performed just before usage, recording the corresponding calibration of the biosensor once the electrochemical biosensor is prepared. The previous modification stages (H_2_O_2_-activation, AA polymerization, and electrodeposition of PtNPs) can be performed in advance, as it was demonstrated that PtNPs-PAA-aSPCEs have a high stability and can be stored at room temperature for up to at least 3 months [29].

To test the applicability of this electrochemical biosensor for the determination of glucose in real samples, a number of possible chemicals that might usually be present within complex biological or food fluids were examined by amperometry. In this regard, mannitol, sucrose, urea, fructose, citric acid, and dehydroascorbic acid (DHA) were added to a buffered solution to obtain a final 100 µM concentration for each, and they exhibited no amperometric response (data not shown). However, the ascorbic acid (100 µM) did affect the amperometric response by increasing the current signal. The interference of ascorbic acid is very common in electrochemical sensing based on PtNPs for oxidative potentials [14]. Nevertheless, this interference can be easily removed by the addition of the enzyme ascorbate oxidase into the sample solution [27,44], as the product of the enzymatic catalysis, dehydroascorbic acid (DHA), does not produce any electrochemical signal at the working electrode potential. The introduction of an ascorbate oxidase spatula (reusable immobilized enzyme) into the medium in the presence of ascorbate quickly reverted the current signal to the original level (current before the addition of ascorbic acid), with the advantage of not contaminating the reaction medium. Moreover, the signal for 100 mM glucose was the same in both the absence and presence of the interferences selected. These results indicate that GOx-PtNPs-PAA-aSPCEs exhibit excellent selectivity properties for glucose measurement. However, for an accurate quantification of glucose in samples containing ascorbate, a previous enzymatic conditioning using ascorbate oxidase is indispensable. 

Some of the analytical merits of the electrochemical glucose biosensor GOx-PtNPs-PAA-aSPCE were compared to other electrochemical glucose biosensors found in the literature (Table 1). Because of the great number of electrochemical glucose sensors reported, we focused on a representative number of amperometric biosensors containing the glucose oxidase enzyme. In general, the GOx-PtNPs-PAA-aSPCEs developed herein showed an improved sensitivity and limit of detection compared with the majority of biosensors shown in Table 1. Only the biosensors published in the literature [23,38,41] showed a slightly higher sensitivity in the same order of magnitude. In addition, the GOx-PtNPs-PAA-aSPCE biosensor has a good linear range (from micromolar to millimolar) in contrast with other biosensors, such as those reported in the literature [45,46]. The limit of detection is also lower than the majority of the biosensors exposed here, except for those reported in [23,35,41] and [45], which have slightly lower limits of detection in the same order of magnitude (micromolar). In addition, the potential applied for GOx-PtNPs-PAA-aSPCE is lower than that used for other first-generation glucose biosensors based on the measurement of H_2_O_2_ at positive potentials (for example in [2,37,45]).

Finally, the developed electrochemical biosensor was used to determine the glucose concentration in real samples (three commercial fruit juices and a plant culture medium (Gamborg’s B-5)), and the data obtained were compared with a classical spectrophotometric method (commercial glucose assay kit, used as a reference method; Table 2). The results showed good accuracy in the measurement of glucose with recoveries between 94.8 and 105.3%. Therefore, it demonstrated the good performance of the electrochemical biosensor in the analysis of real samples. Some signal was observed in Gamborg’s B-5 by the glucose assay kit, despite the fact that it contains no glucose, according to its chemical composition [31], probably as a result of any interference. Instead, GOx-PtNPs-PAA-aSPCE did not show any electrochemical signal in the Gamborg’s B-5 sample, as expected.

## 4. Conclusions

The electrochemical glucose biosensor GOx-PtNPs-PAA-aSPCE has been successfully validated for the amperometric determination of glucose in real samples in terms of sensitivity, limit of detection, linear range, selectivity, repeatability, and reproducibility, as well as its application to glucose quantification in juices and a plant cell culture medium. The platinum nanoparticulate onto the PAA modified aSPCE electrode resulted in a good environment for the immobilization of the enzyme glucose oxidase, with optimum values of 30 mg · mL^−1^ for enzyme loading and 1 h for the immobilization of GOx. The glucose concentration in the real samples was satisfactorily measured and compared with a standard spectrophotometric method, obtaining recoveries between 94.8 and 105.3%. Analytical figures of merit were compared to those obtained with other enzymatic glucose biosensors, highlighting an enhanced sensitivity and limit of detection in most cases. Therefore, the biosensor based on GOx has been demonstrated to be cost-effective, has a good analytical performance, and can be easily prepared. The modification protocol exposed here could open up a promising alternative in the development of new electrochemical biosensors based on other oxidase enzymes.

## Figures and Tables

**Table 1:** Electroanalytical parameters of glucose determination for a variety of enzymatic biosensors based on GOx.

Electrode	Potential (V)	Sensitivity (µA · mM^−1^ · cm^−2^)	LOD (µM)	Linear Range (mM)	Ref.
GOx/Fe_3_O_4_@Au/SPCE	0.38 (vs. Ag)	2.52	100.0	0.2–9	[2]
Au_NC_Pt_Nf_/GOx	0.15 (vs. Ag)	33.66	-	0.01–2	[14]
GOx-PoPD/PtNPs/PVF+ClO_4_^−^/Pt	0.6 (vs. Ag/AgCl)	17.40	18.0	0.06–9.64	[22]
GOx/PtNP/PANI/Pt	0.56 (vs. SCE)	96.10	0.7	0.01–8	[23]
MRGO/PtAuNPs/Ch-GOx/PDDA- modified hybrid electrode	0.6 (vs. Ag/AgCl)	17.85	1.0	0.01–8	[35]
Pt/rGO/P3ABA-SPCE	0.5 (vs. Ag)	22.01	44.3	0.25–6	[37]
GOx/Naf/MnO_2_-GNR/SPCE	0.5 (vs. Ag/AgCl)	56.32	50.0	0.1–1.4	[38]
GOD/Pt/MWNT-PANI	0.55 (vs. SCE)	127.77	1.0	0.003–8.2	[41]
PAA-VS-PANI/GPL-FePc/GOx-CH	0.3 (vs. Ag)	18.11	6.4	1–20	[45]
GOx-GO-SH-Au-SPE	−0.55 (vs. Ag)	3.17	319.4	3–9	[46]
Paper-based maskless enzymatic sensor	−0.1 (vs. Ag)	9.52	120.0	0.3–15	[47]
Gr/PANI/AuNPs/GOx	−0.4 (vs. Ag/AgCl)	20.32	100.0	0.2–11.2	[48]
GOx-PtNPs-PAA-aSPCE	0.2 (vs. Ag)	42.70	7.6	0.02–2.30	This work

GOx/Fe_3_O_4_@Au/SPCE—GOx on Au seeds decorated on magnetic core Fe_3_O_4_ nanoparticles immobilized on a screen-printed carbon electrode. Au_NC_Pt_Nf_/GOx—bimetallic nanocoral Au decorated with Pt nanoflowers. GOx-PoPD/PtNPs/PVF+ClO_4_^−^/Pt—glucose biosensor based on the immobilization of glucose oxidase in an electropolymerized poly(*o*-phenylenediamine) film on a platinum nanoparticles-polyvinylferrocenium modified electrode. GOx/PtNP/PANI/Pt—GOx immobilized on a Pt nanoparticle/polyaniline hydrogel heterostructure. MRGO/PtAuNPs/Ch-GOx/PDDA-modified hybrid electrode—glucose oxidase onto multi-layer reduced graphene oxide sheets decorated with platinum and gold flower-like nanoparticles. Pt/rGO/P3ABA-SPCE—platinum/reduced graphene oxide/poly(3-aminobenzoic acid) nanocomposite film on a screen-printed carbon electrode. GOx/Naf/MnO_2_-GNR/SPCE—GOx and nafion on manganese dioxide nanoparticles decorated on graphene nanorribbons. GOD/Pt/MWNT-PANI—glucose oxidase immobilized on platinum nanoparticles onto the surface of a multi-wall carbon nanotube-polyaniline nanocomposite. PAA-VS-PANI/GPL-FePc/GOx-CH—polyacrylic acid-based conducting hydrogel incorporated with iron phthalocyanine functionalised graphene nanoplatelets and glucose oxidase. GOx-GO-SH-Au-SPE—GOx-decorated thiolated graphene modified gold-sputtered screen-printed electrode. Gr/PANI/AuNPs/GOx—graphene/polyaniline/Au nanoparticles/glucose oxidase biocomposite modified screen-printed electrode.

**Table 2:** Determination of glucose in real samples using a reference spectrophotometric method (commercial glucose assay kit based on GOx) and the electrochemical biosensor GOx-PtNPs-PAA-aSPCE. Each sample was analyzed in triplicate. To remove the ascorbate present in the samples, an ascorbate oxidase spatula was previously introduced into the cell, and the measurements were recorded after 20 min.

Sample	GOx-PtNPs-PAA-aSPCE (g/100 mL)	Glucose Assay Kit (g/100 mL)	Recovery (%)
Orange juice	1.83 ± 0.12	1.93 ± 0.01	94.8
Pineapple juice	3.96 ± 0.01	3.76 ± 0.18	105.3
Peach juice	1.14 ± 0.17	1.13 ± 0.06	100.8
Gamborg’s B-5	-	0.07 ± 0.01	-

## Supplementary Materials

The following are available online at https://www.mdpi.com/1424-8220/20/16/4489/s1: Figure S1: Influence of GOx concentration and immobilization time on the sensitivity of the electrochemical glucose biosensor GOx-PtNPs-PAA-aSPCE; Figure S2: Anodic LSV responses of the different electrode modification steps in the absence and presence of glucose; Figure S3: XPS O1s and N1s spectra for the PAA-SPCE and PAA-aSPCE; Figure S4: XPS C1s and N1s spectra for the PAA-aSPCE and the PtNPs-PAA-aSPCE; Figure S5: Current response vs. time of the GOx-PtNPs-PAA-aSPCE upon successive additions of glucose; Figure S6: Relative sensitivity obtained by the measurement of glucose using the electrochemical glucose biosensor GOx-PtNPs-PAA-aSPCE after different storage times; Table S1: Values of the equivalent circuit elements obtained by fitting the experimental results from Figure 3.
