# Supplementary material for: Glucose Biosensor Based on Disposable Activated Carbon Electrodes Modified with Platinum Nanoparticles Electrodeposited on Poly(Azure A)
Source: Sensors (Basel). 2020 Aug 11;20(16):4489. doi: 10.3390/s20164489 (PMC7472169; doi:10.3390/s20164489)
Supplement: Supplementary file 1 [file sensors-20-04489-s001.pdf]

SUPPLEMENTARY MATERIAL

Glucose Biosensor Based on Disposable Activated Carbon Electrodes Modified with Platinum Nanoparticles Electrodeposited on Poly(Azure A)

Francisco Jiménez-Fiérrez [1], María Isabel González-Sánchez [1], Rebeca Jiménez-Pérez [1], Jesús Iniesta [2] and Edelmira Valero [1,*]

[1] Department of Physical Chemistry, Higher Technical School of Industrial Engineering, University of Castilla-La Mancha, Campus Universitario s/n, Albacete 02071, Spain; Francisco.JFierrez@uclm.es (F.J.-F.); MIsabel.Gonzalez@uclm.es (M.I.G.-S.); Rebeca.Jimenez@uclm.es (R.J.-P.)

[2] Department of Physical Chemistry and Institute of Electrochemistry, University of Alicante, 03690, San Vicente del Raspeig, Alicante, Spain; Jesus.Iniesta@ua.es

* Correspondence: Edelmira.Valero@uclm.es

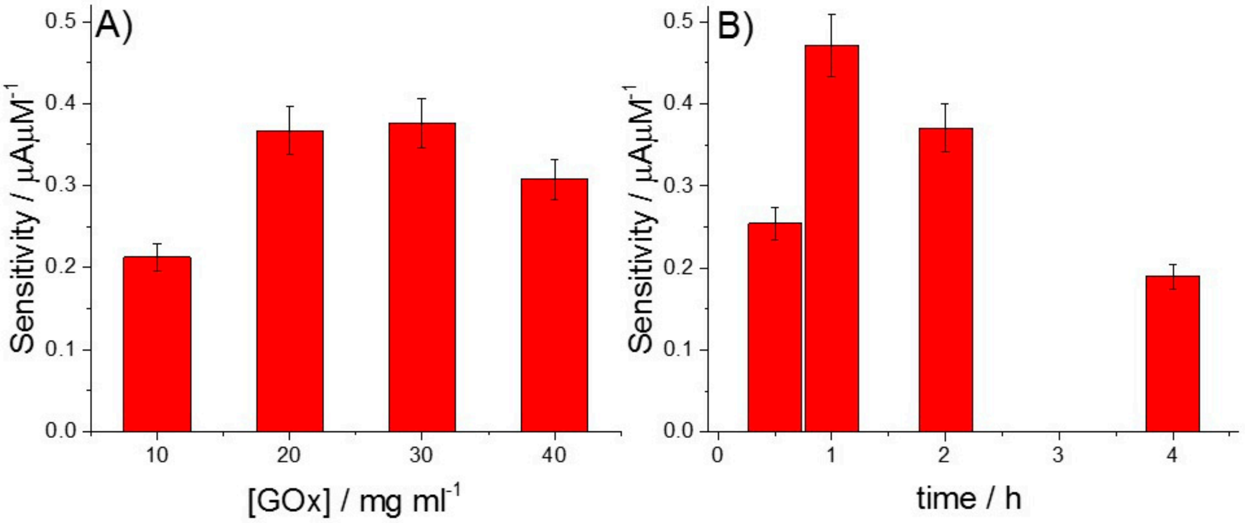

Figure S1. (A) Influence of GOx concentration for the preparation of the electrochemical glucose biosensor (10, 20, 30 and 40 mg·ml^{-1}) on the sensitivity of the electrochemical glucose biosensor GOx-PtNPs-PAA-aSPCE. The enzyme solution was dropped cast during 2 h. (B) Influence of the immobilization time on the sensitivity of the electrochemical glucose biosensor GOx-PtNPs-PAA-aSPCE (0.5, 1, 2, and 4 h). The GOx concentration was 30 mg·ml^{-1}. The electrochemical response to glucose was measured by chronoamperometry at 0.2 V in 100 mM PB solution pH 7 under stirring conditions. The sensitivity was calculated from three different calibration plots performed in a concentration range between 10 and 120 µM of glucose.

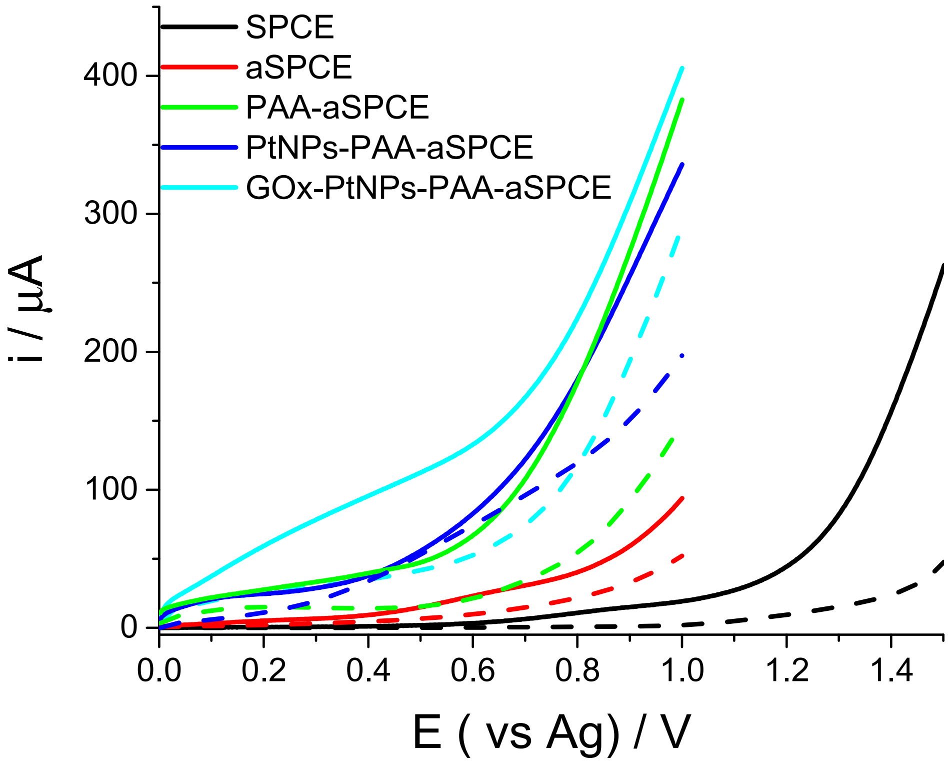

Figure S2. Anodic LSV responses of the different electrode modification steps in the absence (dashed lines) and the presence of 50 mM glucose (solid lines). The measurements were performed in 0.1 M PB.

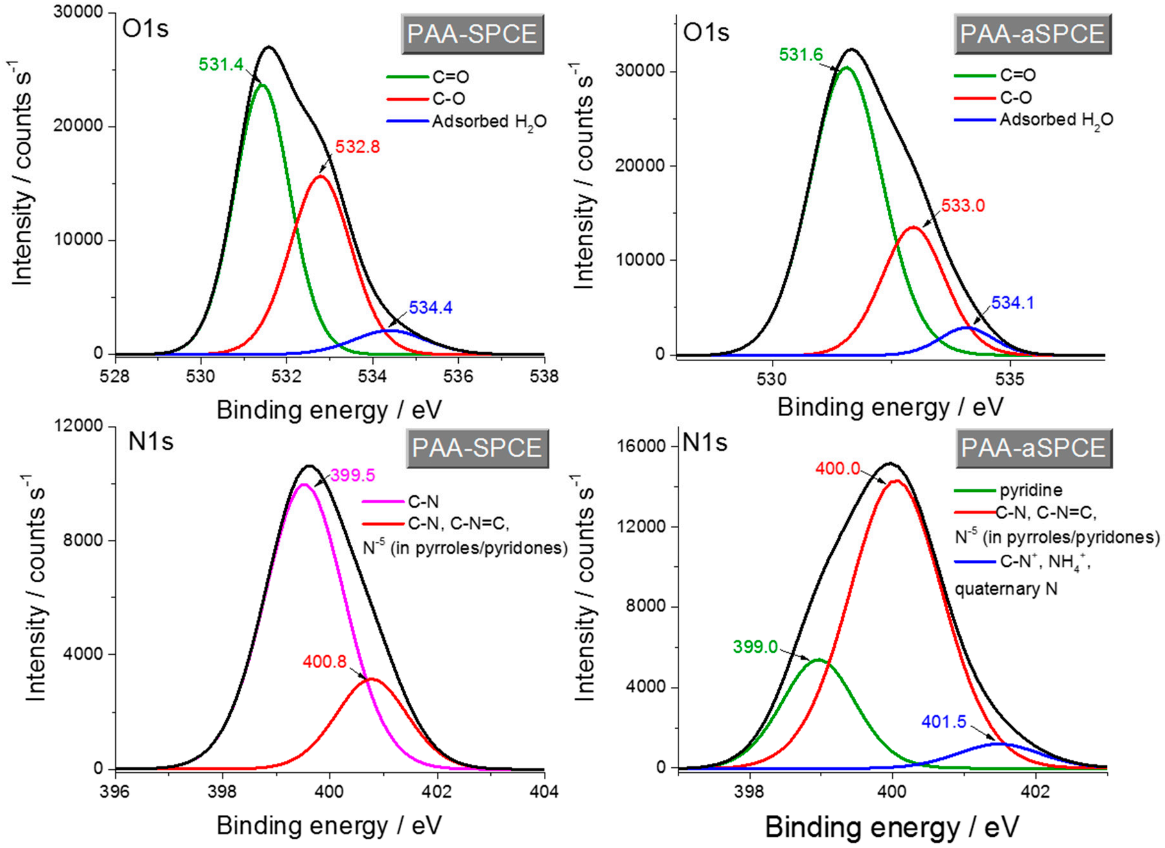

Figure S3. XPS O1s and N1s spectra for the PAA-SPCE and PAA-aSPCE and the deconvolution of their binding peaks linked to functional groups.

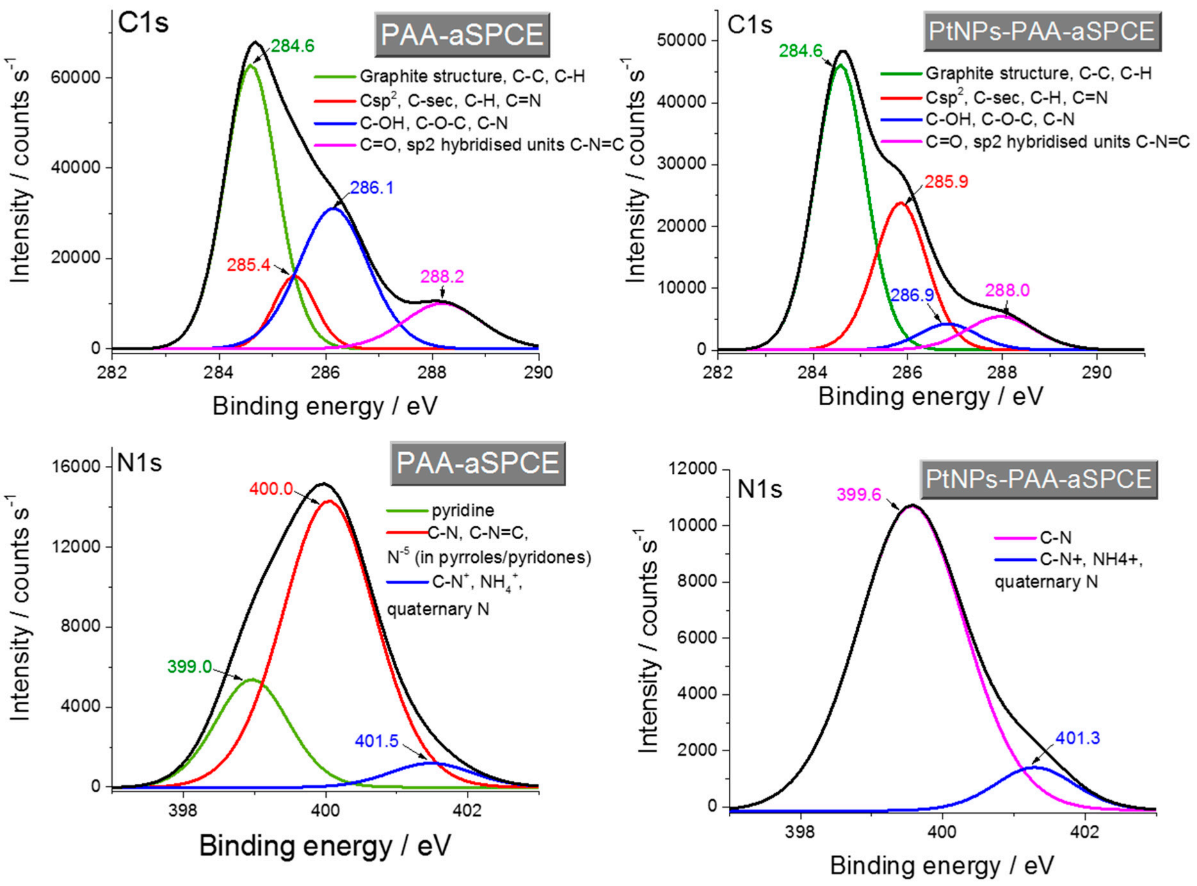

Figure S4. XPS C1s and N1s spectra for the PAA-aSPCE and the PtNPs-PAA-aSPCE and deconvolution of their binding peaks linked to functional groups.

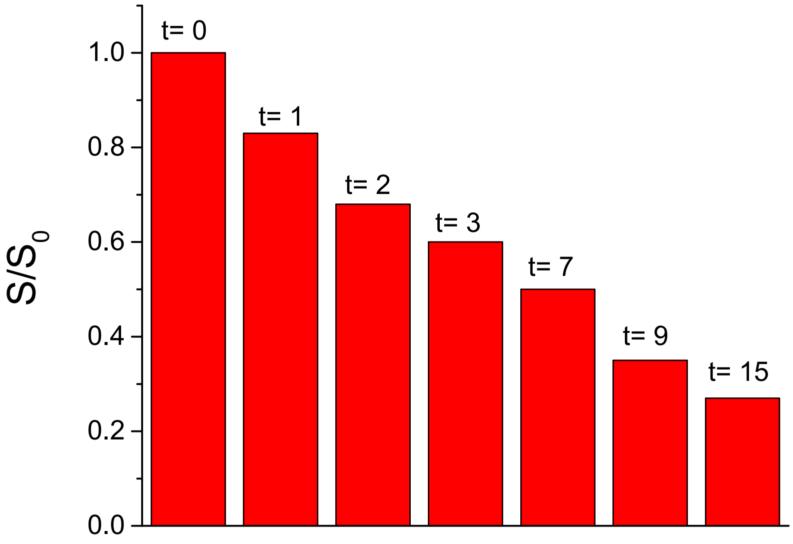

Figure 5. Relative sensitivity (S/S_0) obtained by the measurement of glucose using the electrochemical glucose biosensor GOx-PtNPs-PAA-aSPCE after different storage times (0, 1, 2, 3, 7, 9 and 15 days), being S_0 the sensitivity obtained at t = 0. Sensitivities were calculated performing the calibration plots in a concentration range between 10 and 120 mM of glucose.
